# Comparative analysis of the organelle genomes of *Aconitum carmichaelii* revealed structural and sequence differences and phylogenetic relationships

**DOI:** 10.1186/s12864-024-10136-1

**Published:** 2024-03-08

**Authors:** Rongxiang Zhang, Niyan Xiang, Changjiang Qian, Shuwen Liu, Yuemei Zhao, Guiyu Zhang, Pei Wei, Jianfeng Li, Tao Yuan

**Affiliations:** 1 School of Biological Science, Guizhou Education University, Guiyang, 550018 China; 2 School of Ecology and Environment, Tibet University, Lhasa, 850000 China; 3 Key Laboratory of Development and Utilization of Biological Resources in Colleges and Universities of Guizhou Province, Guizhou Education University, Guiyang, 550018 China; 4 State Key Laboratory of Hybrid Rice, Laboratory of Plant Systematics and Evolutionary Biology, College of Life Sciences, Wuhan University, Wuhan, 430072 China

**Keywords:** *Aconitum carmichaelii*, Chloroplast genome, Mitochondrial genome, Ranunculaceae family, Phylogenetic analysis

## Abstract

In this study, we conducted an assembly and analysis of the organelle genomes of *Aconitum carmichaelii*. Our investigation encompassed the examination of organelle genome structures, gene transfer events, and the environmental selection pressures affecting *A. carmichaelii*. The results revealed distinct evolutionary patterns in the organelle genomes of *A. carmichaelii*. Especially, the plastome exhibited a more conserved structure but a higher nucleotide substitution rate (NSR), while the mitogenome displayed a more complex structure with a slower NSR. Through homology analysis, we identified several instances of unidirectional protein-coding genes (PCGs) transferring from the plastome to the mitogenome. However, we did not observe any events which genes moved from the mitogenome to the plastome. Additionally, we observed multiple transposable element (TE) fragments in the organelle genomes, with both organelles showing different preferences for the type of nuclear TE insertion. Divergence time estimation suggested that rapid differentiation occurred in *Aconitum* species approximately 7.96 million years ago (Mya). This divergence might be associated with the reduction in CO_2_ levels and the significant uplift of the Qinghai-Tibet Plateau (QTP) during the late Miocene. Selection pressure analysis indicated that the dN/dS values of both organelles were less than 1, suggested that organelle PCGs were subject to purification selection. However, we did not detect any positively selected genes (PSGs) in Subg. *Aconitum* and Subg. *Lycoctonum*. This observation further supports the idea that stronger negative selection pressure on organelle genes in *Aconitum* results in a more conserved amino acid sequence. In conclusion, this study contributes to a deeper understanding of organelle evolution in *Aconitum* species and provides a foundation for future research on the genetic mechanisms underlying the structure and function of the *Aconitum* plastome and mitogenome.

**Supplementary Information:**

The online version contains supplementary material available at 10.1186/s12864-024-10136-1.

## Background

*Aconitum* is a perennial herb belonging to the Ranunculaceae family, encompassing approximately 400 species worldwide [1]. In China alone, there are roughly 170 species of *Aconitum*, primarily found in the Qinghai-Tibet Plateau (QTP) and its surrounding regions, with additional populations in northern provinces [2]. As a medicinal plant, *Aconitum* is highly toxic due to its elevated levels of diterpenoid alkaloids (DAs), which can induce damage to the central nervous system, heart, and gastrointestinal tract [3]. Consequently, indiscriminate use of *Aconitum* poses a significant risk to human health and safety [4]. According to the Shennong Bencao Jing, *Aconitum* finds its primary use in treating conditions such as heart failure, rheumatism, arthralgia, bruises, strokes, and paralysis [5]. Moreover, it exhibits notable anti-inflammatory and analgesic properties, with compounds like 3-acetylaconitine, hyperaconitine, lappaconitine, and crassicauline A being employed in non-narcotic clinical medications.

Due to its valuable medicinal properties, the wild population of *Aconitum* has suffered from exploitation and habitat destruction by humans, resulting in a significant decline in its population. However, distinguishing these species based on morphology alone has proven to be challenging, which in turn poses difficulties for conservation efforts. Therefore, there is an urgent need for a molecular method to differentiate *Aconitum* species. Hong et al. [6] noted that even the application of phylogenetic analyses utilizing four intergenic spacer regions and nuclear markers did not effectively address series classification. Plastomes are independent organelles within plant cells, possessing complete sets of genomes, relatively conservative genetic compositions and structures, and a greater number of mutation sites. These structural characteristics have led to the widespread use of plastomes in distinguishing and studying the evolution of plant species [7, 8]. Simultaneously, protein coding genes (PCGs) in mitogenomes also exhibit high conservation, enhancing the ability to resolve phylogenetic relationships within taxa [9, 10].

The absence of *Aconitum* plants nuclear genomic data obviously hinders the taxonomic identification and phylogenetic study of these plants. Therefore, the sequencing of the organelle genome has become a very convenient and effective method for this. Unfortunately, the only mitogenome of the *Aconitum kusnezoffii* [11] has been reported for *Aconitum* spp. plants so far. In this study, we conducted de novo assembly and analysis of chloroplast and mitochondrial genomes from the medicinal plant *Aconitum carmichaelii*. Comparative genomic analysis was used to determine the phylogenetic position and genomic characteristics of *A. carmichaelii* within the Ranunculaceae family. This analysis provides the essential theoretical foundation for molecular identification and the phylogenetic study of species within the Ranunculaceae family.

## Results

### Characterization of the organelle genomes

**Fig. 1:**
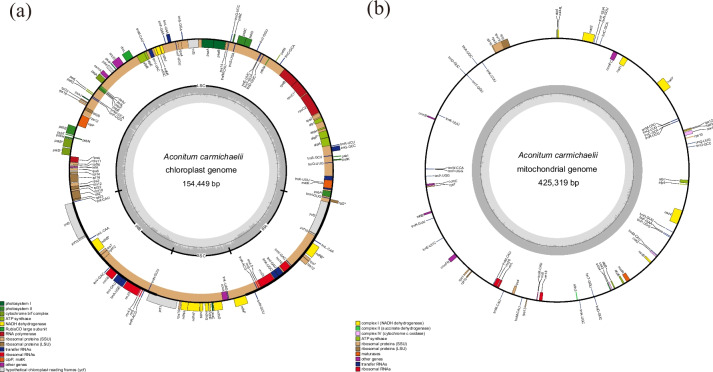
Genome map of the plastome and mitogenome of *A. carmichaelii*. Genes belonging to functional groups that are color-coded on the circle as transcribed clock-wise (outside) and transcribed counter clock-wise (inside). The darker grey in the inner circle represents the GC content, while the lighter grey represents the AT content. **A** Plastome of *A. carmichaelii*; **B** Mitogenome of *A. carmichaelii*

The plastome of *A. carmichaelii* exhibited the typical quadripartite structure and had a total length of 154,449 bp (Fig. 1a). The Large Single Copy (LSC) region (89,059 bp) and the Small Single Copy (SSC) region (16,946 bp) were separated by two Inverted Repeats (IRs) regions (24,222 bp), and the plastome had a GC content of 38.1% (Table S2). It contained 125 genes, including 81 PCGs, 36 tRNA genes, and 8 rRNA genes. Among the PCGs, 41 were associated with photosynthesis, while 71 were related to self-replication, with one pseudogene, *ψrpl16*, being identified (Table 1). The mitogenome of *A. carmichaelii* assembled as a single loop chromosome of 425,319 bp with a GC content of 46.7% (Fig. 1b). It comprised 68 genes, consisting of 34 PCGs, 30 tRNA genes, and 4 rRNA genes. Notably, the nicotinamide adenine dinucleotide dehydrogenase gene *nad5* and *nad9* was absent from the mitogenome (Table 2).

**Table 1:** List of genes in the plastome of *A. carmichaelii*

Category	Group genes	Name of genes
Transcription and translation	Large subunit of ribosome (LSU)	*rpl2*, *rpl14*, *ψrpl16*, *rpl20*, *rpl22*, *rpl23*, *rpl33*, *rpl36*
	Small subunit of ribosome (SSU)	*rps2*, *rps3*, *rps4*, *rps7* (× 2), rps8, *rps11*, *rps12* (× 2), *rps14*, *rps15*, *rps16*, *rps18*, *rps19*
	RNA polymerase	*rpoA*, *rpoB*, *rpoC1*, *rpoC2*
	Translational initiation factor	*InfA*
	rRNA genes	*rrn4.5* (× 2), *rrn5* (× 2), *rrn16* (× 2), *rrn23* (× 2)
	tRNA genes	*trnA-UGC* (× 2), *trnC-GCA*, *trnD-GUC*, *trnE-UUC*, *trnF-GAA*, *trnM-CAU*, *trnG-GCC*, *trnG-UCC*, *trnH GUG*, *trnI-CAU*, *trnI-GAU* (× 2), *trnK-UUU*, *trnL-CAA* (× 2), *trnL-UAA*, *trnL-UAG*, *trnM-CAU*, *trnN-GUU*(× 2), *trnP-UGG*, *trnQ-UUG*, *trnR-ACG* (× 2), *trnR-UCU*, *trnS-GCU*, *trnS-GGA*, *trnS-UGA*, *trnT GGU*, *trnT-UGU*, *trnV-GAC* (× 2), *trnV-UAC*, *trnW*-*CCA*,*trnY*-*GUA*
Photosynthesis	Photosystem I	*psaA*, *psaB*, *psaC*, *psaI*, *psaJ*
	Photosystem II	*psbA*, *psbB*, *psbC*, *psbD*, *psbE*, *psbF*, *psbH*, *psbI*, *psbJ*, *psbK*, *psbL*, *psbM*, *psbN*, *psbT*, *psbZ*
	NADH oxidoreductase	*ndhA*, *ndhB* (× 2), *ndhC*, *ndhD*, *ndhE*, *ndhF*, *ndhG*, *ndhH*, *ndhI*, *ndhJ*, *ndhK*
	Cytochrome b6/f complex	*petA*, *petB*, *petD*, *petG*, *petL*, *petN*
	ATP synthase	*atpA*, *atpB*, *atpE*, *atpF*, *atpH*, *atpI*
	RubiscoCO large subunit	*rbcL*
	ATP-dependent protease subunit gene	*clpP*
Other genes	Maturase	*matK*
	Envelop membrane protein	*cemA*
	Subunit Acetyl- CoA-Carboxylate	*accD*
	c-type cytochrome synthesis gene	*ccsA*
Unknown	Conserved Open reading frames	*ycf1* (× 2), *ycf2* (× 2), *ycf3*, *ycf4*, *ycf15* (× 2)

The Values denoted as 'x2' represent the presence of two copies of the gene

ψ is represented as a pseudogene

**Table 2:** Gene composition in the mitogenome of *A. Carmichaelii*

Group of genes	Name of genes
ATP synthase	*atp1*, *atp4*, *atp6*, *atp9*
Cytochrome c biogenesis	*ccmB*, *ccmC*, *ccmFC*, *ccmFN*
Ubichinol cytochrome c reductase	*cob*
Cytochrome c oxidase	*cox1*, *cox2*, *cox3*
Maturases	*matR*
Transport membrane protein	*mttB*
NADH dehydrogenase	*nad1*, *nad2*, *nad3*, *nad4*, *nad4l*, *nad5*, *nad6*, *nad7*
Large subunit of ribosome	*rpl2*, *rpl10*, *rpl16*
Small subunit of ribosome	*rps1*, *rps3*, *rps4*, *rps7*, *rps10*, *rps11*, *rps12*, *rps13*
Succinate dehydrogenase	*sdh3*, *sdh4*
Ribosomal RNAs	*rrn5*, *rrn18*, *rrn26*
Transfer RNAs	*trnA-CGC*, *trnA-UGC*, *trnC-GCA*, *trnD-GUC*, *trnE-UUC*, *trnF-GAA*, *trnG-GCC*, *trnH-GUG*, *trnI-CAU*, *trnK-UUU*, *trnM-CAU*, *trnN-GUU*, *trnP-UGG*, *trnQ-UUG*, *trnR-UCG*, trnS-GCU, *trnT-GGU*, *trnW-CCA*, *trnY-GUA*

### Codon usage and ENc-GC3s analysis

**Fig. 2:**
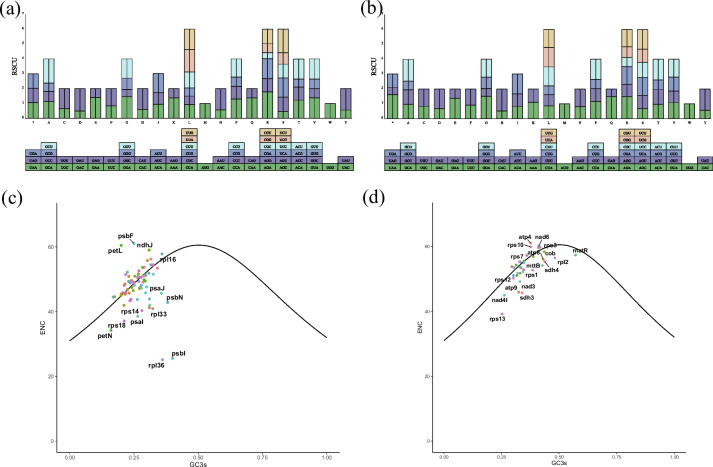
Relative synonymous codon usage and ENC plotted against GC3s based on PCGs of *A. carmichaelii* organelle genes. **A** the chloroplast genes. **B** the mitochondrial gene. **C** the plastome PCGs. **D** the mitogenome PCGs. The solid line indicates the expected curve of positions of genes when the codon usage is merely determined by the GC3s composition

The codon usage of *A. carmichaelii* is presented in Fig. 2 and Table S3. The plastome and mitogenome PCGs of *A. carmichaelii* utilize 61 codons to encode 20 amino acids. Among these, leucine (11.05%/10.13%) is the most frequently encoded amino acid, while tryptophan (1.71%/1.42%) is the least. This finding contrasted with earlier observations regarding the amino acid coding in *Aconitum*'s plastome, as reported by Xia et al. [12]. Additionally, we conducted an analysis of the RSCU and the codon base composition of protein-coding gene sequences in *A. carmichaelii*. In both organelle genomes, 28 codons have RSCU values greater than 1, 34 codons have RSCU values less than 1, and two codons have RSCU values equal to 1. Among the 64 codons in the plastome, 16 end with A, U, G, or C. Moreover, among the codons with RSCU values greater than 1, 13 end with A, 14 end with U, 1 ends with G, while none end with C (Fig. 2). A similar pattern was observed in the mitogenome, among codons with RSCU values greater than 1, 11 end with A, 15 end with U, 1 ends with G, and 1 ends with C (Table S3). These results suggested that codons in the *A. carmichaelii* organelle genome predominantly terminate with A or U, consistent with prior findings in the plastome of *Aconitum* species as reported by Meng et al. [13].

To investigate the relationship between nucleotide composition and codon bias in the organelle genome of *A. carmichaelii*, we analyzed the CAI, CBI, FOP, ENC and GC content at the third codon position (GC3s) in PCGs of both the plastome and mitogenome (Tables S4 and 5). The results reveal that ENC values for plastome PCGs range from 25 to 61 (Fig. 2c), whereas those for mitogenome PCGs range from 39 to 61 (Fig. 2d). Only three plastome PCGs exhibit a high codon bias (ENC < 35), while none of the mitogenome PCGs have ENC values below 35, indicated that these genes did not display a strong codon preference. Most genes fall below the expected ENC curve, with only a few lying above it, suggested that conditional mutations might have a limited influence on codon preference formation.

### SSRs and repeat elements in *A. carmichaelii* organelle genomes

Simple sequence repeats (SSRs) have been widely utilized as molecular markers in population genetic studies, encompassing both intraspecific and interspecific polymorphisms [14] and population genetics [15], owing to their high polymorphism and co-dominant inheritance. To further enhance our analysis, we conducted a comprehensive examination of microsatellite sequences within both the plastome and mitogenome. The results showed that 39 SSR sequences were present in the plastids, of which 34 SSR were located in the LSC region, 2 in the SSC region, and 3 in the IRs region, while 31 microsatellite sequences were present in the mitogenome, of which 22 were located in the intergenic region, and 9 were located in the genic region (Table S6). we observed the occurrence of mononucleotide, dinucleotide, and trinucleotide repeats. In contrast, the mitogenome exhibited five different types of repeats, albeit without the presence of hexanucleotide repeats. Among the single nucleotide repeats, polyadenine (polyA) and polythymine (polyT) repeats were predominant (Table S7), while tandem guanine (G) or cytosine (C) repeats were relatively scarce. These findings align with prior research on chloroplast SSRs [13]. In terms of quantity, the higher number of SSR sequences within the plastome renders it more suitable for conducting population genetics studies.

Repeated sequences, both in the reverse and forward orientations, have played a crucial role in shaping the size and structure of plant organelle genomes. They have been instrumental in driving genomic rearrangements, facilitating repetitive sequence-mediated recombination, as well as insertion and deletion events [16, 17]. We conducted an analysis to identify matching repeat sequences in both the plastome and mitogenome of *A. carmichaelii* using BLASTn. In the plastome, the majority of repeats were found to have a size ranging from 39 to 72 bp. Notably, we only identified two repeat sequences that exceeded 100 bp in length, with the exception of two reverse-repeating IR regions (Fig. S1a and Table S7). In contrast, the mitogenome of *A. carmichaelii* exhibited a more extensive repertoire of repeated sequences. Specifically, we identified six repeat sequences exceeding 100 bp in length, with the longest repeat sequence spanning an impressive 13,097 bp (Fig. S1b and Table S7). These findings suggested that the mitogenome of *A. carmichaelii* contains a greater abundance of repetitive sequences compared to its plastome counterpart. This increased presence of repetitive sequences in the mitogenome might be associated with a higher frequency of gene recombination events and chromosomal rearrangements within the mitogenome.

### Comparative plastome analysis

**Fig. 3:**
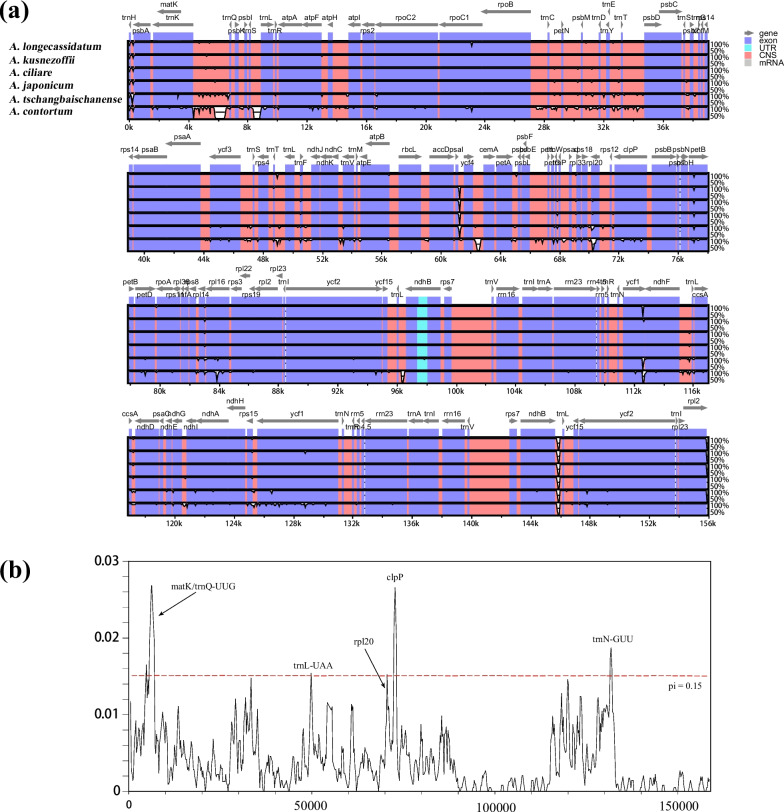
Comparative plastome analysis. **A** Comparison of seven plastomes using *A. carmichaelii* annotation as a reference. The vertical scale indicates the percentage of identity, ranging from 50 to 100%. The horizontal axis indicates the coordinates within the plastome. Genome regions are color-coded as exons, introns and conserved non-coding sequences (CNS). **B** Sliding-window analysis on the plastomes for seven *Aconitum* species. X-axis: position of the midpoint of a window; Y-axis: nucleotide diversity (Pi) of each window

To investigate variability among plastome sequences within the genus *Aconitum*, we employed the plastome of *A. carmichaelii* as our reference. We compared the plastome sequences of seven *Aconitum* species using mVISTA software (Fig. 3a). The results revealed a high degree of conservation within the plastome of *Aconitum*. Notably, the LSC and SSC regions exhibited greater differentiation than the IR regions. This divergence in the LSC and SSC regions can be attributed to copy correction through gene conversion following mutations in the IR region, a phenomenon documented by Khakhlova and Bock [18]. Furthermore, it is worth noting that non-coding regions displayed a higher level of conservation when compared to coding regions. In summary, our study identified four significantly differentiated regions (Pi ≥ 0.05) within the plastome of *Aconitum*: *matK-trnQ-UUG*, *trnL-UUA*, *rpl20*, and *trn-GUU* (Fig. 3b). Among these regions, the *rpl20* gene emerged as a hotspot, aligning with previous research findings [19]. These identified hotspot regions hold the potential to serve as valuable molecular markers and barcodes for *Aconitum*, laying the foundation for future phylogenetic analyses and species identification efforts.

### Comparative mitogenome analysis

**Fig. 4:**
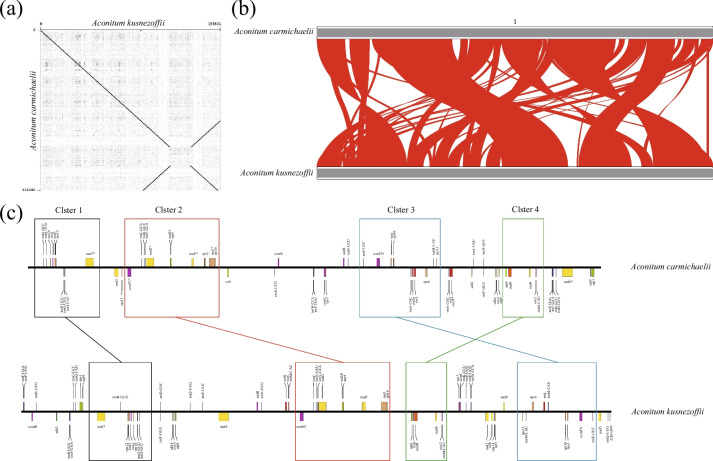
Collinearity block analyses of organelle genomes between two *Aconitum* species. **A** collinearity regions of plastomes between two *Aconitum* species. **B** collinearity regions of mitogenomes between two *Aconitum* species. **C** Conserved gene blocks in the mitogenome of two *Aconitum* species

Collinearity block analysis is a common approach for discerning evolutionary relationships among closely related species at the genome level. In light of this, we conducted a collinearity block analysis to investigate structural disparities within the mitogenomes of *Aconitum* species. Our analysis aimed to identify homologous regions between the two organelle genomes. Notably, the plastomes of *A. carmichaelii* and *A. kusnezoffii* displayed a high degree of collinearity (Fig. 4a). On the other hand, we identified over 50 collinearity blocks exceeding 1,000 bp in length within the mitogenomes of *A. carmichaelii* and *A. kusnezoffii* (Table S8). This observation suggested that the mitogenomes of these two *Aconitum* species exhibit a more intricate collinearity structure (Fig. 4b).

In summary, it is apparent that the mitogenomes of *Aconitum* species feature a more complex structure compared to their plastomes. Additionally, our examination of the positions of orthologous genes between the two *Aconitum* species revealed structural rearrangements that disrupted gene clusters within the mitogenomes (Fig. 4c). However, it is worth noting that certain gene clusters remain conserved in *Aconitum* species, including gene cluster 1, gene cluster 2, gene cluster 3, and gene cluster 4, as illustrated in Fig. 4c.

### Identification of TEs and gene transfer

The plastome and mitogenome of *A. carmichaelii* contain numerous TE fragments with a combined length of 17,110 bp and 27,971 bp, constituting 11.08% and 6.58% of the total length of the plastome and mitogenome, respectively (Table 3). These TE fragments can be categorized into two main classes: DNA transposons and retrotransposons. Notably, DNA transposons make up a substantial portion of the plastome, accounting for 10,948 bp (63.99%), while retrotransposons are more prominent in the mitogenome, comprising 14,981 bp (53.56%). Furthermore, differences in the types of TEs are observed between the plastome and mitogenome. For instance, Penelope elements were exclusively detected in the mitogenome.

**Table 3:** Comparation of TEs in *A. carmichaelii* organelle genomes

Repeat Class	Plastome		Mitogenome	
	Fragments	Lengths	Fragments	Lengths
Integrated Virus	2	134	8	1152
Caulimoviridae	2	134	8	1152
Multicopy gene	9	536	12	736
tRNA	9	536	12	736
Transposable Element	155	16,440	256	26,083
DNA transposon	98	10,948	91	7001
EnSpm/CACTA	20	2081	15	1056
Harbinger	4	601	4	243
Helitron	28	3263	25	2236
Mariner/Tc1	1	77	1	79
MuDR	26	3373	37	2783
hAT	16	1290	8	464
LTR Retrotransposon	48	4847	131	14,981
Copia	31	3699	54	6350
Gypsy	16	1050	75	8489
Non-LTR Retrotransposon	8	598	34	4101
L1	7	529	32	3948
Penelope	\	\	1	53
Naiad/Chlamys	\	\	1	53
SINE	1	69	1	100
SINE2/tRNA	1	69	\	\
Total	166	17,110	276	27,971
Ratio	/	11.08%	/	6.58%

The ratio was obtained by dividing the transposon sequence length by the genome length

Previous studies have reported the presence of plastome gene residues in the mitogenome, suggested significant sequence transfer between these two organelles [20]. To identify potential gene transfer fragments between the plastome and mitogenome, we conducted a search using BLASTn and obtained a total of 19 fragments (Table S9). The longest fragment had a length of 1,280 bp, and the total length of these sequences was 5,675 bp. After annotation, we identified several highly homologous plastome-derived PCGs in the mitogenome, including *psbB*, *psbG*, and *rps12*, referred to as mitochondrial plastid DNAs (MTPTs) (Fig. S2). However, we did not find any intact mitogenome-derived PCGs in the plastome. These results suggested that intracellular DNA has been transferred from the plastome to the mitogenome in *A. carmichaelii*.

### Phylogenetic analysis and divergence time estimation

**Fig. 5:**
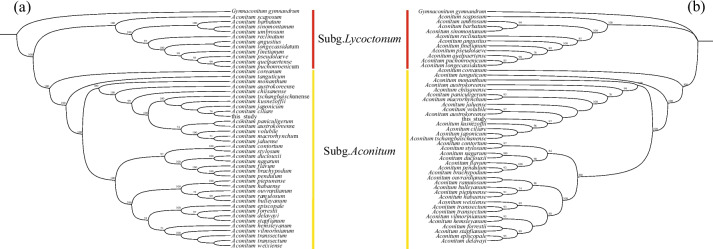
Phylogenetic tree of *Aconitum* species. **A** The phylogenetic tree based on 79 protein-coding genes. **B** The phylogenetic tree based on whole plastome

In the present study, we selected two datasets, the whole plastome and 79 PCGs, from among the plastomes of 47 *Aconitum* species and one outgroup plastome to construct the phylogenetic relationships among *Aconitum* species. The topology of the ML tree constructed using the 79 PCGs was essentially consistent with that of the whole-genome ML tree (Fig. 5). However, the support rates of the ML trees constructed based on different datasets varied. Based on the analysis of the 79 PCGs, the phylogenetic results indicated that *Aconitum* species could be classified into two major groups: Subg. *Aconitum* and Subg. *Lycoctonum* (bootstrap support: 100%). This finding agreed with previous research [12, 21]. Additionally, the whole-genome phylogenetic results also strongly supported the division of *Aconitum* into Subg. *Aconitum* and Subg. *Lycoctonum*. *A. carmichaelii* was identified as the sister species to *A. japonicum*, *A. tschangbaischanense*, *A. ciliare*, and *A. kusnezoffii*, indicated a close relationship among them.

The estimated divergence time indicited that Subg. *Aconitum* and Subg. *Lycoctonum* diverged approximately 7.96 Mya, while the ancestor of *A. carmichaelii* and *A. austrokoreense* diverged around 0.51 Mya (Fig. S3). Subsequently, they diverged from *A. kusnezoffii* and *A. ciliare* approximately 0.47 Mya. The results of this study contribute valuable genome resources for the phylogenetic analysis of *Aconitum* and offer a reference for the phylogenetic study of the Ranunculaceae family and other related research endeavors.

### Selective pressures analysis

Because the two subgenera of *Aconitum* diverged as early as 7.96 Mya, they subsequently followed distinct evolutionary trajectories. We hypothesized that Subg. *Aconitum* and Subg. *Lycoctonum* might exhibit differences in their evolutionary rates. Therefore, we utilized the yn00 module in PAML v4.9j software [22] to compute the dN and dS substitution rates for 73 shared plastome genes and 31 shared mitogenome genes in pairs.

**Fig. 6:**
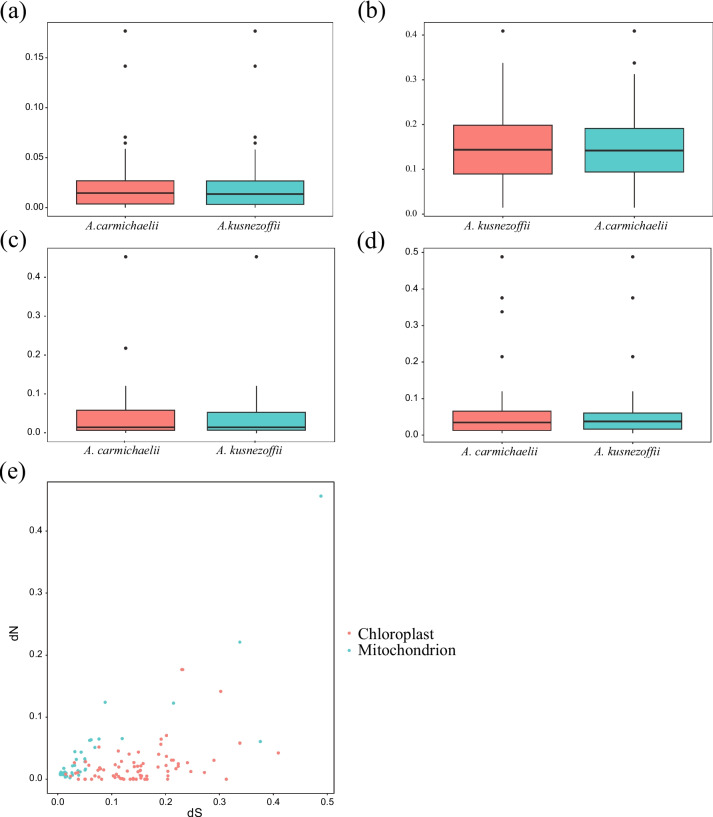
Variation in sequence divergence across species and organelles. **A** Comparison of dN values across two *Aconitum* plastomes. **B** Comparison of dS values across two *Aconitum* plastomes. **C** Comparison of dN values across two *Aconitum* mitogenomes. **D** Comparison of dS values across two *Aconitum* mitogenomes. **E** Comparison of dN and dS values across organelles

The results revealed that the dS value of the plastome in two *Aconitum* species exceeded the dN value (Figs. 6a and b), whereas the dN value of the mitogenome in these species was comparable to the dS value (Figs. 6c and d). Importantly, the relatively high dS suggested that the *Aconitum* plastome is presently in a stable state, undergoing evolution at a slower pace. Furthermore, both *Aconitum* organelles exhibited dN/dS values below 1, provided evidence for purifying selection acting on PCGs within the *Aconitum* organelles. In summary, the mutation rate of mitogenome genes in *Aconitum* species was lower than that of plastome genes, particularly in terms of synonymous mutations (Fig. 6e). We further conducted a Mann–Whitney U test on the dN and dS values of plastome PCGs and mitogenome PCGs, revealing that the dS and dN values of plastome PCGs were significantly higher than those of mitogenome PCGs (*p*-value of dS = 2.909e-07; *p*-value of dN = 0.02137). These findings demonstrated that, although some genes in both organelle genomes exhibit similar mutation rates, plastome genes tend to mutate at a faster rate than genes in the mitogenome. Positive selection signals are often interpreted as indications that a species has adapted to its environment [23]. Consequently, we designated Subg. *Aconitum* as foreground branches and conducted a positive selection analysis of 73 shared plastome PCGs using the branch-site model. However, it is regrettable that no potential PSGs were identified in this study.

## Discussion

The plastome of *A. carmichaeli* was found to be consistent with previous studies on *Aconitum* species in terms of size, structure, and gene order, indicated the conservation of plastomes within *Aconitum* species [12]. *Aconitum* species exhibit a high degree of diversity, and many of them share similar plant morphology and medicinal root sites, making it challenging to identify them based solely on morphological characteristics. Molecular markers can enhance the accuracy of identification. Previous research has identified highly divergent regions, including *trnK-UUU-trnQ-UGG*, *ndhJ-ndhK*, *psbH-petB*, *trnA-UGC-trnI-GAU*, *psbD*, *clpP*, and *ycf1*, through a comparison of the plastomes of three *Aconitum* medicinal plants [12]. However, the number of molecular markers available for *Aconitum* plastomes was relatively limited. In this study, we identified four highly divergent regions (*matK-trnQ-UUG*, *trnL-UUA*, *trn-GUU* and *rpl20*) by comparing the genome sequences of seven sequenced *Aconitum* species. The findings of this study have contributed to the expansion of available molecular markers for *Aconitum*. These newly discovered regions with high mutation rates, both within genes and intergenic regions, could serve as valuable molecular markers for conducting phylogeographic and population genetics studies within the *Aconitum* genus.

The utilization of genomic codons varies significantly among different species and organisms, making codon usage preference a crucial evolutionary feature of the genome. Among these preferences, RSCU serves as a fundamental indicator, where RSCU > 1 indicates a high codon usage frequency, RSCU < 1 denotes low frequency, and RSCU = 1 implies no codon usage preference [24]. This preference is believed to result from a combination of natural selection, species-specific mutations, and genetic drift. In this study, the codons in the organelle genome of *A. carmichaelii* were observed to predominantly end in A/U, aligning with prior reports on *Aconitum germplasm* [13] (Meng et al., 2018), which indicated that there was A/U bias in the third codon of *A. carmichaelii* gene. A/U preference may be related to RNA structure and stability. Because A and U form more stable base pairs, RNA molecules containing more A/U base pairs may contribute to RNA stability and secondary structure formation [25], and Codons with A/U bias are more common in specific protein structural elements [26]. Additionally, the ENC values serve as an indicator of codon bias for the 20 amino acids in the Open Reading Frame (ORF) region of PCGs, with values ranging from 20 to 61. Values near 20 indicate a preference for a single synonymous codon, while those near 61 suggest equal usage of each synonymous codon. In this investigation, most PCGs in both the plastome and mitogenome of *A. carmichaeli* were found to be below the expected ENC curve, indicated that natural selection was the primary factor shaping codon usage preferences [27]. This trend was especially pronounced in genes related to photosynthesis and respiration, subject to intense environmental selection pressures due to variable environmental conditions. However, not all genes associated with photosynthesis and respiration exhibited ENC values falling on the expected curve, suggesting that mutations played a minor role in shaping codon preferences. These findings underscored that natural selection is the dominant force driving codon usage preferences in *A. carmichaeli*, particularly in crucial genes involved in photosynthesis and respiration [28].

Pseudogenes have played a significant role in the regulation of gene expression and the evolution of species [29]. Previous studies have consistently identified several pseudogenes, namely *ycf15*, *rps16*, *infA*, *rps19*, and *ycf1*, as commonly present in the *Aconitum* plastome [12, 13, 30]. However, in this particular study, only one pseudogene, *rpl16*, was detected, and it was located within the LSC-IRB junction region. The location of *rpl16* in the LSC-IRB junction region has been shown to place it at the boundary of each region of the plastome. Duplication events occurring at the boundaries of the IRs and the SSC/LSC regions have resulted in genetic incompleteness within the IRs region. This phenomenon is commonly referred to as the "boundary effect" [31]. Based on the findings of this study, it is evident that the types of pseudogenes present in different species vary, indicating that the occurrence of pseudogenes differs among *Aconitum* species.

Phylogenetic analysis revealed that the topology of the ML tree, constructed using 79 PCGs, was largely consistent with that of the whole-genome ML tree. At the intra-generic level, all *Aconitum* species were classified into Subg. *Aconitum* and Subg. *Lycoctonum* clades, aligning with previous studies [12, 21]. Notably, the branch support based on the PCGs dataset was higher than that derived from the complete plastome dataset. These findings indicated that phylogenetic relationships among *Aconitum* species, based on plastome PCGs, provided more efficient results than using whole genomes alone. In our present study, we observed that *Aconitum* species underwent rapid divergence primarily around 7.96 Mya. A recent study, based on Late Miocene global sea surface temperature reconstructions, suggested that a reduction in CO_2_ levels around 7 Mya triggered a significant global cooling event known as the Late Miocene cooling [32, 33]. Concurrently, research by Chen et al. [34] has demonstrated that the QTP and the Xining Basin experienced substantial uplift during the Late Miocene, leading to the acceleration of aridification in the Asian interior. Consequently, we propose that the reduction in CO_2_ levels and the uplift of the QTP collectively contributed to the rapid divergence of *Aconitum* species during the Late Miocene, approximately 7.96 Mya.

The dN, dS, and dN/dS ratio serve as indicators of a gene's response to natural selection [35]. A dN/dS value greater than 1 suggests positive selection, while a value less than 1 indicates purification or negative selection. A dN/dS value of 1 signifies neutral selection [36]. In this study, we observed that the dS values of the plastomes in the two *Aconitum* species exceeded the dN values, indicated the current stability and slow evolution rate of *Aconitum* plastomes. Furthermore, the dN/dS values for both *Aconitum* organelles were less than 1, provided evidence for the purification selection acting on *Aconitum* organelle PCGs. We also noted that the plastome exhibited a faster NSR compared to the mitogenome, as evidenced by the comparison of dN and dS values across all PCGs in these organelles. A Mann–Whitney U test revealed that the dS and dN values of plastome PCGs were significantly higher than those of mitogenome PCGs. These distinct genetic features between the two organelles may be attributed to their different genomic repair mechanisms [37]. Furthermore, we did not detect PSGs in Subg. *Aconitum*, which suggested a consistent environmental selection pressure among *Aconitum* species. These results align with the NSR findings, indicated ongoing purification selection acting on plastome genes within *Aconitum* species. This study contributed to a deeper understanding of organelle evolution in *Aconitum* plants and laid the groundwork for further investigations into the genetic mechanisms underlying the structure and function of plant plastomes and mitogenomes.

## Materials and methods

### Taxon sampling and sequencing

*A. carmichaelii* specimens were collected from Yongle Town, Nanming District, Guiyang City, Guizhou Province, China, and were subsequently cultivated under natural conditions in the garden of Guizhou Education University. The samples were identified by Prof. Changjiang Qian from Guizhou Education University, and voucher specimens were deposited at the Guizhou Education University, China. Total genomic DNA from their fresh leaves was isolated by CTAB [38]. The extracted total genomic DNA was used to construct DNA libraries and sequenced on the Illumina and Pacbio platforms of GrandOmics (Wuhan, China), respectively. For Illumina sequencing, after the library was constructed (with an insert size of 350 bp), Qubit 3.0 was used for preliminary quantification, and after the library was diluted, Agilent 2100 was used for quality control of the library inserts, and after the size of the inserts met the expectation, q-PCR was used to accurately quantify the effective concentration of the libraries to ensure the quality of the libraries. Finally, sequencing was performed on the DNBSEQ-T7 platform. For PacBio sequencing, Megaruptor 3 was used to interrupt 8 μg of DNA for genomic fragmentation, and the interruptions were purified using AMPure PB magnetic beads. 15 Kb fragment libraries constructed by the PacBio Sequel II platform were used to perform multiple rounds of sequencing on individual fragments to improve accuracy. Finally, SMRTlink v11.0, the official software of PacBio, was used to perform quality control statistics on the output data, and valid data were finally obtained.

### Genome assembly, and annotation

The de novo assembly of next-generation sequencing (NGS) reads was conducted using the default settings of GetOrganelle v1.7.5.3 software [39], which successfully extracted the plastome. To obtain a complete and highly accurate mitogenome of *A. carmichaelii*, we employed Canu v2.1.1 software to assemble PacBio HiFi reads with the Canu-hifi parameter [40]. The final plastome and mitogenome were refined using NGS reads with Pilon v1.23 [41]. Plastome annotation was performed using GeSeq [42], while the mitogenome was annotated using references such as *Aconitum kusnezoffii* (NC_053920.1), *Pulsatilla dahurica* (NC_071219.1), *Pulsatilla cernua* (NC_068018.1), and *Coptis omeiensis* (OP466724.1 and OP466725.1). All tRNAs were predicted using tRNAscan-SE software [43] rRNAs were annotated using BLASTN. Finally, Geneious Prime [44] was used to manually correct all annotation errors and OGDRAW [45] was utilized for the visualization of both the plastome and mitogenome.

### Codon usage and ENc-GC3s analysis

In this study, we employed CodonW v1.4.4 software [46] for the analysis of relative synonymous codon usage (RSCU) and Geneious software [44] for the analysis of GC content. In addition, we analyzed the codon adaptation index (CAI), codon deviation index (CBI), optimal codon frequency (FOP) parameters using the Galaxy online website (https://galaxyproject.org/). Effective number of codons (ENC) plots are commonly used to assess codon usage patterns in genes. The relationship between ENC and GC3s was visualized using R scripts available at (https://github.com/taotaoyuan/myscript). Predicted ENC values that lie on or above the expected curve can indicate that codon usage is primarily influenced by G + C mutations. However, if the natural selection or other factors are at working, the predicted ENC values will fall below the expected curve [47].

### Comparison of complete plastome

The plastomes of 6 reported *Aconitum* species were loaded from the NCBI website, which are, *A. longecassidatum* (NC_035894.1), *A. ciliare* (NC_031420.1), *A. japonicum* (KT820670.1), *A. tschangbaischanense* (NC_066973.1), *A. contortum* (NC_038098.1), *A. kusnezofi* (MK782811.1). CGView software was used to evaluate the plastome structures of the seven plants [48]. The mVISTA [49] was used to compare the plastomes of the seven *Aconitum* species in Shuffle-LAGAN mode, with annotation of *A. carmichaelii* as a reference. Sliding window analysis was conducted to determine the nucleotide diversity of the plastome using DnaSP v5 [50], with 200 bp of step size and 600 bp window length.

### Identification of homologous sequences and transposable elements

We utilized the Gepard software [51] to identify potential structural rearrangements by aligning the plastomes of *A. carmichaelii* and *A. kusnezoffii*. The collinearity regions of the plastome between *A. carmichaelii* and *A. kusnezofi,* and among mitogenomes of seven *Aconitum* species plastomes were identified using TBtools [52] with a matching rate of ≥ 80% and an E-value of ≤ 1e-5. These collinearity regions were visualized using the RIdeogram R package [53] with default parameters. Repetitive sequences within the plastome and mitogenome were identified and visualized using TBtools with an E-value ≤ 1e-5. Transposable elements (TEs) in the organelle genomes were identified using the CENSOR web server [54] with 'Viridippantae' as a reference sequence.

### Phylogenomic analysis

Due to the scarcity of mitogenomic data, we were unable to employ for mitochondrial data to reconstruct the phylogenetic relationships of the genus *Aconitum*. Instead, we downloaded 47 plastomes from NCBI (https://pubmed.ncbi.nlm.nih.gov/) to reconstruct the phylogenetic relationships of the genus *Aconitum* (Table S1). Firstly, we extracted a total of 79 protein-coding genes (PCGs) using PhyloSuite v1.2.2 [55], aligned their CDS sequences with MAFFT v7.490 [56], and removed poorly aligned portions using the 'automated1' parameter in TrimAl v1.4.1 [57]. We then constructed a concatenated matrix with FASconCAT-G v1.04 [58] and used it to build a phylogenetic tree. Secondly, we utilized the entire plastomes of 48 *Aconitum* species to construct another phylogenetic tree. Next, the best models for the 79 PCGs datasets and 48 whole plastomes datasets were determined using ModelFinder [59]. Additionally, we inferred the maximum likelihood (ML) tree with IQ-TREE [60] using the best models and performed 5000 ultrafast bootstrapping.

The MCMCTree package of PAML v4.9j [22] was employed to analyze the divergence times. Fossil calibration point sources primarily included the paleobiodb database (https://paleobiodb.org/) and the Timetree5 website (http://www.timetree.org/), and one fossil calibration point was selected: Time of differentiation in the genera *GymnAconitum* and *Aconitum* (23.20 to 29.61 Mya). Finally, the ChiPlot online website [61] was used for visualizing the results.

### Selective pressures analysis

To compare the nucleotide substitution rate (NSR) of mitogenomes between the two *Aconitum* species, we used *Pulsatilla dahurica* (mitogenome: NC_072536.1; plastome: MK860685.1) as a reference to calculate the NSR of protein-coding genes (PCGs) in the two *Aconitum* mitogenomes. We extracted 79 shared plastome PCGs and 16 mitogenome PCGs using PhyloSuite v1.2.2 [55] and calculated the synonymous (dS) and nonsynonymous (dN) substitution rates using the KaKs_Calculator [62] with the yn00 model. To assess whether there is a difference in selection pressure between Subg. *Aconitum* and Subg. *Lycoctonum* in *Aconitum*, we set 11 Subg. *Aconitum* species as the foreground branch. The dN/dS ratio was calculated using the Codeml package in PAML v4.9j software [22]. Simultaneously, the presence of positively selected genes (PSGs) was determined, and the effective *P*-value (*P* < 0.05) was obtained through a Chi-square test on the likelihood ratio test (LRT) value. Positive selection sites were determined using the BEB method. Candidate PSGs were defined as having *p* < 0.05 and ω > 1.

## Conclusions

In this study, we present the plastome and mitogenome of *A. carmichaelii*, with sizes of 154,449 bp and 425,319 bp, respectively. The plastome contains 125 genes, while the mitogenome contains 68 genes. Codon usage analysis revealed a preference for codons ending in A/T bases. ENc-map results suggested that mutations might play a minor role in shaping codon preferences. We identified 39 and 31 SSRs in the plastome and mitogenome, respectively. These SSRs hold potential for the development of molecular markers. Notably, the variants in the IRs exhibited higher conservation compared to the LSC and SSC regions. Additionally, four highly differentiated regions (Pi ≥ 0.05) in the plastome of *Aconitum* (*matK-trnQ-UUG*, *trnL-UUA*, *rpl20*, and *trn-GUU*) could serve as molecular markers for species identification and genetic diversity studies. We observed a unidirectional gene transfer between organelles, specifically from plastome to mitogenome. No transfer events from mitogenome to plastome were detected. Both organelle genomes harbor multiple nuclear TEs, with the plastome showed a preference for DNA transposons, while the mitogenome favors reverse transcription transposons. Divergence time estimates suggested that rapid differentiation of *Aconitum* species occurred around 7.96 Mya. This divergence might be attributed to the reduction of CO_2_ levels and the uplift of the QTP during the Late Miocene around 7.96 Mya. The results of selection pressure analysis indicated that the PCGs in both types of *Aconitum* organelles are under purifying selection (dN/dS < 1). Furthermore, plastome PCGs exhibit a higher NSR compared to mitogenome PCGs. This study significantly enriched the genetic resources of *Aconitum* spp. and established a robust scientific foundation for the development of molecular markers, species identification, and phylogenetic studies within the genus *Aconitum*.

### Supplementary Information

**Supplementary Material 1.**

**Supplementary Material 2.**

**Supplementary Material 3.**

**Supplementary Material 4.**

**Supplementary Material 5.**

**Supplementary Material 6.**

**Supplementary Material 7.**

**Supplementary Material 8.**

**Supplementary Material 9.**

**Supplementary Material 10.**

## Data Availability

The plastome and mitogenome sequences generated in this study were deposited in GenBank database under accession numbers OR682676 and OR682677.
