# Supplementary material for: Comparative analysis of the organelle genomes of Aconitum carmichaelii revealed structural and sequence differences and phylogenetic relationships
Source: BMC Genomics. 2024 Mar 8;25:260. doi: 10.1186/s12864-024-10136-1 (PMC10921738; doi:10.1186/s12864-024-10136-1)
Supplement: Supplementary file 10 — Supplementary Material 10. [file 12864_2024_10136_MOESM10_ESM.docx]

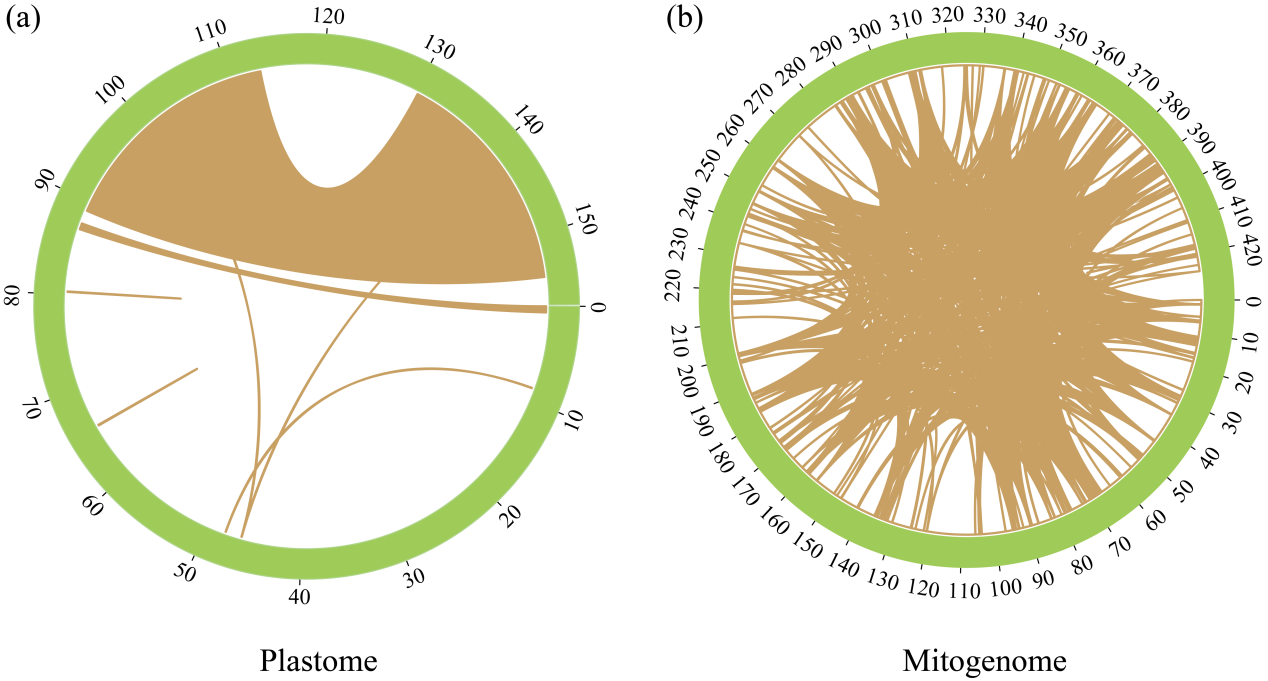


Figure S1. The repeat sequences of plastome and mitogenome. (A) Repeat sequences in plastome. (B) Repeat sequences in mitogenome.


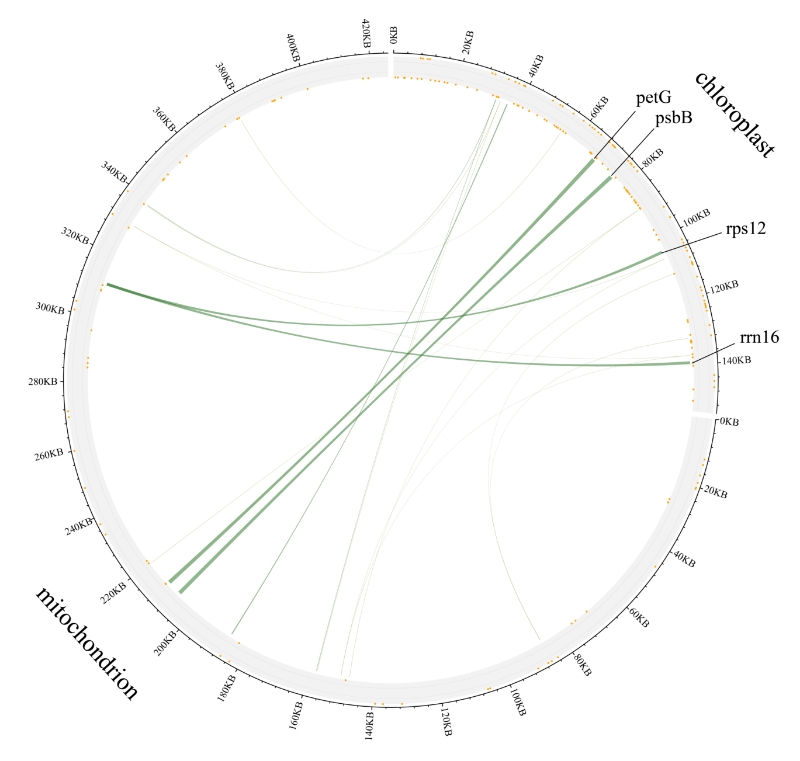


Figure S2. The homologous sequences between plastome and mitogenome.


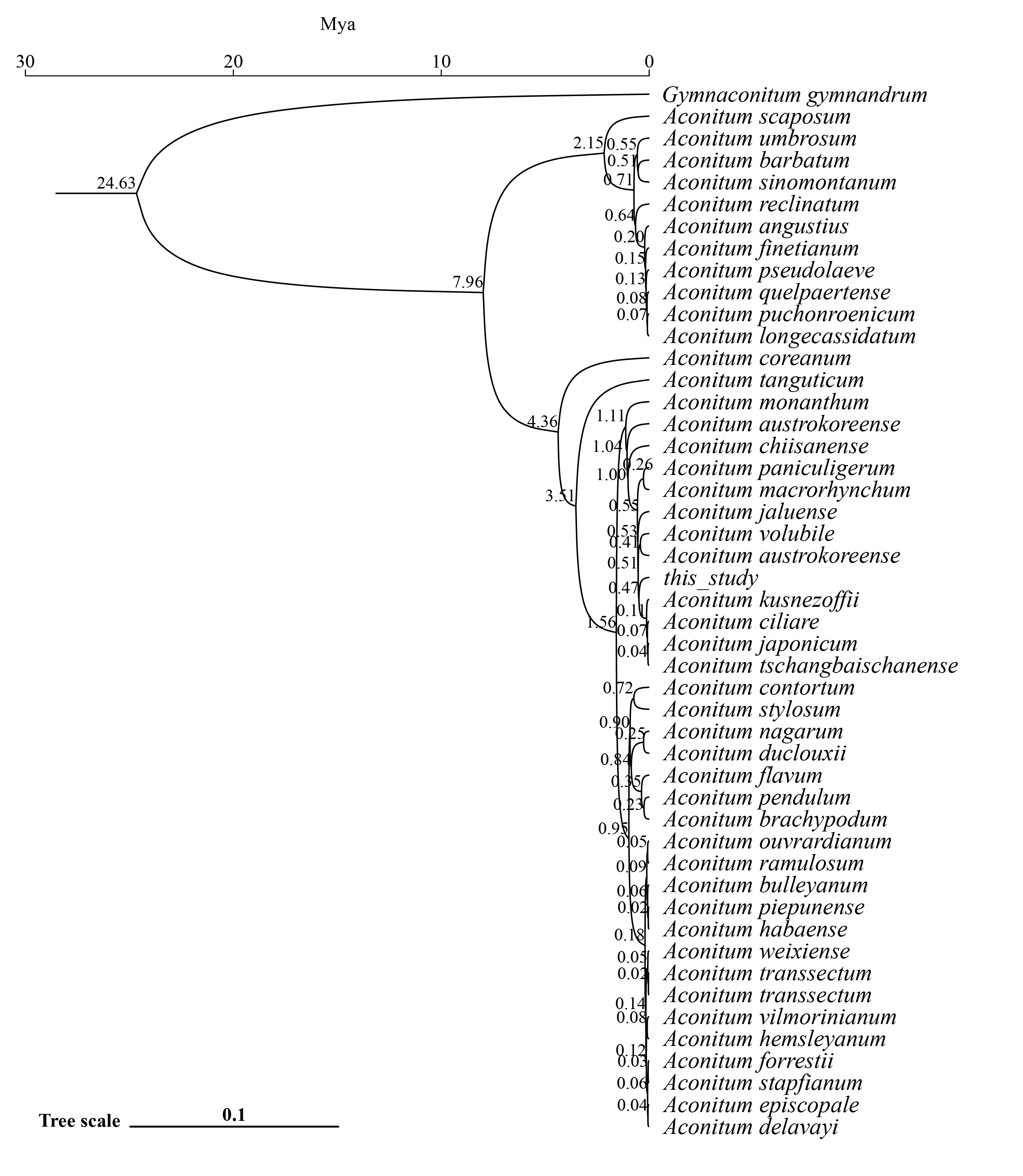


Figure S3. Species divergence time of 48 *Aconitum* species based on 79 PCGs.
